# Microglia Versus Myeloid Cell Nomenclature during Brain Inflammation

**DOI:** 10.3389/fimmu.2015.00249

**Published:** 2015-05-26

**Authors:** Melanie Greter, Iva Lelios, Andrew Lewis Croxford

**Affiliations:** ^1^Institute of Experimental Immunology, University of Zurich, Zurich, Switzerland

**Keywords:** microglia, macrophage, monocyte, dendritic cell, CNS inflammation

## Abstract

As immune sentinels of the central nervous system (CNS), microglia not only respond rapidly to pathological conditions but also contribute to homeostasis in the healthy brain. In contrast to other populations of the myeloid lineage, adult microglia derive from primitive myeloid precursors that arise in the yolk sac early during embryonic development, after which they self-maintain locally and independently of blood-borne myeloid precursors. Under neuro-inflammatory conditions such as experimental autoimmune encephalomyelitis, circulating monocytes invade the CNS parenchyma where they further differentiate into macrophages or inflammatory dendritic cells. Often it is difficult to delineate resident microglia from infiltrating myeloid cells using currently known markers. Here, we will discuss the current means to reliably distinguish between these populations, and which recent advances have helped to make clear definitions between phenotypically similar, yet functionally diverse myeloid cell types.

## Introduction

Most tissues are populated by incredibly diverse and abundant myeloid cells. By contrast, the central nervous system (CNS) harbors comparatively few myeloid cell subsets. This is likely due to the immune privilege and relative isolation enjoyed by the CNS compared to other non-lymphoid tissues such as the gut or the lung, which are continually confronted with foreign entities. In the steady state, the CNS houses several populations of myeloid cells with distinct localizations including perivascular, choroid plexus, and meningeal macrophages/dendritic cells (DCs) and microglia, which are the most abundant (1). Microglia are considered the resident macrophages of the brain given that they are the only myeloid cells present in the CNS parenchyma. Microglia perform both homeostatic and immune-related functions and constitute about 5–20% of all cells in the CNS (2). They use their “ramified” morphology to act as immune sentinels, extending specialized processes, and sampling the local environment for foreign bodies (3, 4). Numerous recent reports have unmasked additional functions for microglia other than being simply the brain’s intrinsic immune system. For example, microglia are also critical for neuronal development, adult neurogenesis, learning-dependent synapse formation, and brain homeostasis (5–7). Microglia are classified as tissue resident macrophages but are clearly ontogenically distinct from other members of the mononuclear phagocyte system (MPS), which includes DCs, monocytes, and macrophages. Microglia originate from primitive macrophages that derive from erythro-myeloid precursors in the yolk sac (8–10). These primitive yolk sac macrophages colonize the developing brain in mice as early as embryonic day 9.5 (8). Throughout adult life microglia remain of embryonic origin in the healthy CNS and maintain themselves locally without any detectable contribution from circulating myeloid progenitors including monocytes. Yolk sac macrophages and microglia precursors in the developing brain express high levels of the fractalkine receptor (CX_3_CR1) and are positive for the integrin alpha M (Itgam, also know as CD11b; macrophage-1 antigen, Mac-1), F4/80, and the macrophage-colony stimulating factor receptor 1 (Csf-1R, CD115) similar to adult microglia as described below (8). Compared to adult microglia, however, microglia precursors are CD45^hi^. The development of microglia is dependent on Csf-1R (CD115), the transcription factors PU.1 and Irf8 but is independent of Myb, which is crucial for the development of hematopoietic stem cells (HSCs) (8, 9, 11, 12). In contrast to microglia, recent adoptive transfer and fate-mapping studies revealed that other macrophage populations are either embryonically derived from definitive hematopoiesis (e.g., alveolar or heart macrophages) or are constantly replaced by circulating monocytes (e.g., dermal or gut macrophages) (10, 13–18). Aside from the unique ontogeny of microglia within the MPS, a clear classification of microglia compared to other tissue macrophages in terms of phenotype and function has been difficult. Only recently, transcriptome and epigenetic analysis identified genes uniquely expressed and regulated by microglia but not by other macrophage populations (19–24). These studies might be useful to classify and distinguish microglia from other myeloid cells.

## Microglia Markers in Steady State

**Figure 1:**
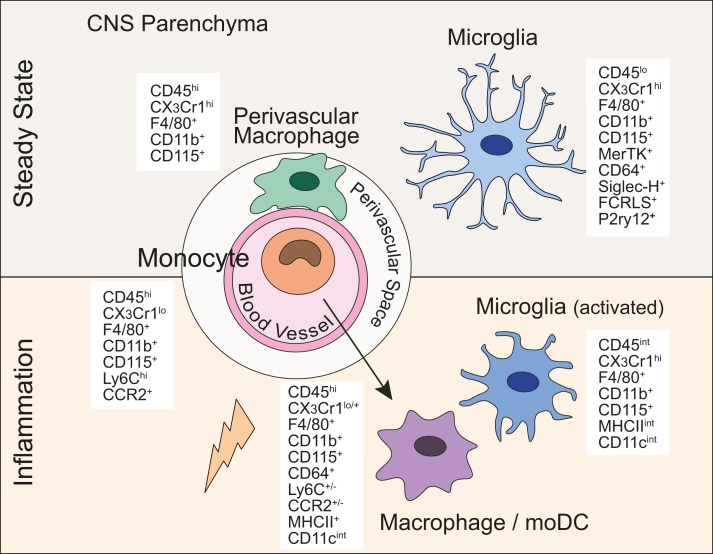
**Central nervous system myeloid cells and their defining lineage markers**. In the steady state and under inflammatory conditions, myeloid cells in the CNS express a diverse, yet overlapping set of markers commonly used to discriminate between MPS members.

In steady state conditions, microglia express surface markers typically present on many other tissue macrophages and/or monocytes such as CD11b, F4/80, Fc-gamma receptor 1 (CD64), and CD115 (Csf-1R), ionized calcium-binding adapter molecule 1 (Iba-1) and proto-oncogene tyrosine-protein kinase MER (MerTK) (Figure 1) (19). In contrast to microglia, which are γ-irradiation resistant, perivascular myeloid cells are replaced by bone marrow (BM)-derived precursors after total-body irradiation and BM transplantation (25–28). However, the exact ontogeny of (non-microglia) myeloid cells associated with the CNS and whether they are also able to maintain themselves locally is, to date, not known (29). These perivascular cells are equipped to present antigen (varying levels of MHCII and CD11c). Whether they represent a homogeneous distinct population or a heterogeneous population of macrophages and/or DCs is not entirely resolved. In the past, a cell expressing F4/80 was deemed to be a macrophage, whereas a cell expressing CD11c was considered a DC. It is clear now that subsets of DCs can also express F4/80 and certain macrophage populations express CD11c. Upon Flt3L treatment, a CD11c^+^MHCII^+^ population in the meninges and choroid plexus expanded, which is indicative of the DC lineage whose development is dependent on Flt3L signaling (27, 28). In addition, a limited number of CD11c^+^ myeloid cells were also described to be in a juxtavascular location in the CNS parenchyma (30). These cells might, however, represent *bona fide* microglia expressing CD11c in certain regions of the brain. Further studies are required to dissect the ontogeny and characterize these elusive myeloid cells associated with the CNS in the steady state.

As common to many other macrophage populations including microglia, most of the CNS-associated myeloid cells also express CD11b, CD115, Iba-1, and F4/80 (31). Therefore, apart from their location, the only available means to unequivocally distinguish microglia from other CNS-resident myeloid populations (CNS-associated macrophages/DCs) and circulating monocytes is by the reduced expression of the common leukocyte antigen CD45, which is readily detectable by flow cytometry. Adult microglia, unlike most other tissue macrophages, constitutively express high levels of the fractalkine receptor CX_3_CR1 (32). A major advance in the microglia field has been the generation of *Cx3Cr1^creER^* mice (32–34). This tamoxifen-inducible Cre-recombinase under the CX_3_CR1 promoter allows for microglia-specific gene targeting. Despite the fractalkine receptor being expressed by monocytes and myeloid precursors in the BM, microglia remain a self-contained population in the CNS and therefore remain targeted long after ceasing of tamoxifen administration, returning the short-lived, circulating myeloid cells to their wild-type origin.

Only recently, gene expression studies have identified surface markers and transcription factors specifically expressed by steady state microglia but not by other macrophage populations or monocytes. These include, for example, sialic acid-binding immunoglobulin-type lectin H (Siglec-H), Fc receptor-like S (Fcrls), and purinergic receptor P2Y G-protein coupled 12 (P2ry12) (20, 21). Furthermore, microglia seem to be the only hematopoietic cell population that specifically expresses Sal-like 1 (Sall1), a transcription factor that plays a crucial role in kidney development (35). While previous studies have reported expression of Sall1 only by stromal cells, in the adult CNS this factor is expressed exclusively by microglia. These aforementioned gene expression studies have compared the transcriptome of microglia to either macrophages derived from the spleen, the lung, the peritoneum, or to monocytes. Whether these microglia core signature markers are also expressed by CNS-associated macrophages/DCs remains to be shown.

## Microglia Markers in Inflammation

In contrast to the healthy brain, during neuro-inflammation the picture becomes far more complicated. A hallmark of microglia is their rapid activation after a CNS insult, resulting in their migration toward injury, proliferation, and their change in morphology. They take on a more “amoeboid” shape with shorter and thicker processes, display increased immunoreactivity for Iba-1 and upregulate CD45. Experimental autoimmune encephalomyelitis (EAE), which is a mouse model for multiple sclerosis (MS), is characterized by infiltration of T cells, monocytes, and neutrophils. Monocytes and their progeny [macrophages/monocyte-derived DCs (moDCs)] are undoubtedly the prevailing cell type in the lesions (see below). However, activated microglia are also clearly detected in the vicinity of the inflammatory lesions. The downregulation of Ly6C by monocytes upon their differentiation adds complexity to the separation of these two distinct cell types based on the commonly used cell surface markers. Additionally, molecules involved in antigen presentation and T cell stimulation, which are barely detectable in steady state microglia, are expressed to some level by microglia already at disease onset and retain expression throughout disease progression. These markers include major histocompatibility complex class II (MHCII), CD11c (also known as integrin alpha X, Itgax), CD80 (B7-1), CD86 (B7-2), and CD40 (36–38). Under these conditions, it is considerably more difficult to distinguish these activated microglia from inflammatory monocyte-derived cells. Similar changes in microglia surface markers have been observed in mouse models of neurodegenerative disease such as Alzheimer’s disease (AD), Parkinson’s disease, and amyotrophic lateral sclerosis (ALS) (20, 31, 39).

A recent study, however, has used differentially expressed chemokine receptors on the surface of microglia and monocytes to distinguish those two myeloid populations and study their function during neuro-inflammation. Microglia were identified by their high expression of CX_3_CR1 whereas infiltrating monocytes, which subsequently differentiate into macrophages/DCs, were defined by their high expression of C–C chemokine receptor type 2 (CCR2), a receptor mediating monocyte recruitment to sites of inflammation. Gene expression profiles from macrophages versus “embryonically derived” microglia at different stages of EAE show that despite some similarities between these inflammatory cell types, microglia exhibit a distinct molecular signature (40). This genetic distinction reflects a different function of resident microglia and infiltrating monocytes under pathological circumstances. While monocyte-derived macrophages seemed to be the effector cell type causing CNS damage, microglia might have a regulatory function and could play a role in tissue repair and homeostasis (40). Another report also showed that monocytes recruited to the CNS in EAE do not acquire microglia-signature genes (21). These studies will unquestionably help attributing unique functions to microglia and CNS-invading myeloid cells in different pathological conditions in the brain.

Whether microglia-specific surface markers and transcription factors alter their expression between steady state and inflammation remains unclear. The microglia-specific ATP receptor P2ry12 was downregulated under inflammatory conditions such as lipopolysaccharide (LPS) systemic injection or in SOD1 mouse-model of ALS (41, 42). On the other hand, P2ry12 and Fcrls continue to be expressed by microglia in EAE but are not expressed by infiltrating monocytes (21). Therefore, further studies need to be undertaken in order to better characterize these “new” phenotypic microglia markers under pathological conditions.

## Monocyte-Derived Microglia

Even though microglia homeostasis is maintained through local self-renewal, under certain conditions circulating precursors can give rise to microglia-like cells. For example, early studies using BM-chimeras showed that up to 10–20% of microglia were reconstituted by donor-derived cells 6-12 months after total-body irradiation and BM transplantation (43). However, this engraftment of BM-derived microglia can only be seen upon blood–brain-barrier (BBB) disruption (e.g. irradiation) and becomes minimal in models where the BBB is unperturbed (e.g. protection of the head during irradiation and parabiosis) (44, 45). This clearly indicates that under steady state conditions, monocytes or BM-derived myeloid progenitors do not infiltrate the CNS parenchyma and thus do not give rise to adult microglia. Similarly, as described above in experimental models of neuro-inflammation, monocytes infiltrate the CNS and differentiate into effector cells resembling phenotypically activated microglia. Despite these similarities, monocyte-derived cells do not persist in the CNS after inflammation has been resolved and thus do not contribute to long-lived microglia (46).

Finally, local administration of ganciclovir to transgenic mice expressing the thymidine kinase of herpes simplex virus under the CD11b promoter (CD11b-HSVTK) leads to a rapid depletion of microglia (47, 48). Subsequently, BM-derived cells enter the CNS and differentiate into long-lived microglia-like cells. Notably, while these cells form a network filling the niche for embryonically derived microglia, they do not obtain a complete microglia phenotype. Monocyte-derived “microglia” in this model show a less-ramified morphology and a higher expression of CD45 compared to yolk sac-derived microglia resembling more activated microglia (48). It has not yet been investigated whether these monocyte-derived “microglia” functionally resemble embryonically derived microglia or whether they acquire microglia-signature genes (Siglec-H, Fcrls, P2ry12) as described above.

Recent studies used a Csf-1R inhibitor to deplete microglia. Upon treatment stop, microglia were repopulated within 1 week (49, 50). These studies showed that new “microglia” were derived from CNS-resident nestin-positive precursors and resembled embryonically derived microglia in response to an inflammatory stimulus. Animals with newly repopulated “microglia” did not display any impairment in behavior, cognition, or motor function compared to control animals (50).

## Monocytes and Monocyte-Derived Cells in CNS Inflammation

Brain inflammation or “encephalitis” invariably results in a reshaping of the myeloid cell populations inhabiting the CNS. An inflammatory response brought on by either infection or autoimmune manifestations results in a rapid increase in blood-derived cellularity to this otherwise dormant site. Despite EAE being fully dependent on T helper cells (51), the vast majority of the inflammatory infiltrate seen in EAE is of myeloid derivation. Two types of monocytes exist including the classical monocytes (Ly6C^hi^CCR2^+^CX_3_CR1^lo^) and the non-classical monocytes (Ly6C^lo^CCR2^−^CX_3_CR1^hi^). Here, we will only discuss Ly6C^hi^ monocytes given that during neuro-inflammation, this is the subset recruited to the brain. Engraftment of phagocytes derived from circulating CCR2^+^ monocytes has also been shown in an AD mouse model (39). Ly6C^hi^ monocytes egress from the BM and cross the BBB in a CCR2-dependent manner (52, 53) followed by their differentiation into macrophages/moDCs and upregulation of a set of cell surface markers (e.g., MHCII, CD11c) expressed on a wide variety of MPS members. Likewise, microglia progressively alter their phenotype to resemble more classically activated macrophages during CNS inflammation, infection, and neuronal or myelin damage (54).

Ly6C^+^ monocytes were shown to migrate into the CNS prior to disease onset and precede the development of paralysis and subsequent clinical manifestations of EAE, when “DC-like” cells are found in abundance in the inflamed tissue (55, 56). This corresponds well with a previous report showing CD205^+^ myeloid cells accumulating in the meninges, choroid plexus, and subpial space of the spinal cord and in perivascular cuffs in demyelinating lesions during acute disease (57). CD11b^+^ DCs within the inflamed CNS were demonstrated to be critical for the propagation of EAE (27, 58, 59). Further phenotypical characterization would be required to demarcate their lineage whether they resemble moDCs or are more similar to classical DCs. Indeed, monocyte-derived antigen presenting cells (APCs) have been shown to be required for optimal priming of T cells in models of infection (60). Current evidence suggests that phenotypically similar macrophages in the CNS can not only contribute to the generation of inflammatory lesions and perform a pathogenic role in the demyelination process but also contribute to regenerative repair mechanisms to resolve inflammation (61, 62). These studies emphasize that distinct functions are attributed to the different subsets of myeloid cells in the course of a CNS inflammation. As such, a complete understanding of cell types based on surface phenotype alone would be of great benefit both in preclinical models of CNS inflammation and also in human patients.

Even with the knowledge we now possess on myeloid cell diversity, it is still commonplace in the literature using animal models of CNS inflammation to use a simplistic CD45^hi^CD11b^hi^ gating strategy to separate CNS infiltrating, blood-derived myeloid cells from CNS-resident, embryonically derived microglia (CD45^low^) (63). Efforts to sort cells using a broad CD45^hi^CD11b^hi^ surface phenotype from within the inflamed CNS will inevitably result in analysis of multiple cell types, lacking any of the desired specificity. Indeed, without the removal of Ly6G^hi^ cells during sorting, a mixed population is inevitable and expression profiles subsequently attributed to moDCs are either confused with, or heavily influenced by, an abundant neutrophil contamination. Even if the effort is taken to remove neutrophils, moDCs at various stages of development will be incorporated. This distinction is increasingly important given that both neutrophils and moDCs have been shown to mediate BBB permeability and demyelination, and that different pathogenic mechanisms are likely active in the two populations during the same inflammation (40, 64).

We know that at least four clearly distinct cell types share this rather non-specific CD45^hi^CD11b^hi^ surface phenotype in an inflamed CNS, namely neutrophils (CD11b^+^Ly6G^+^), monocytes (CD11b^+^Ly6C^hi^CX_3_CR1^low^), and their progeny such as moDCs and/or activated macrophages (Figure 1). The latter two cell types represent most likely the same population with just different names assigned by different studies. Ly6C^hi^ monocytes that have migrated into the CNS can further be subdivided into numerous differentiation stages characterized by the upregulation of CD11c and MHCII, with the concomitant downregulation of Ly6C and CCR2. Upon differentiation and upregulation of MHCII, monocytes are then called moDCs/activated macrophages. Thus, moDCs in the CNS are characterized by the expression of CD11b^+^F4/80^+^MHCII^+^CD11c^int^Ly6C^+/−^. These moDCs/activated macrophages also express CD64 and likely also MerTK, which both are universally expressed by tissue macrophages including microglia (19). Interestingly, it has been shown that monocytes recruited to the CNS during EAE do not express the newly identified microglia markers Fcrls and P2ry12 highlighting again the diverse ontogeny of these cell types and suggesting that the microglia-signature genes are indeed specific to microglia rather than location (CNS) specific (21). The FcεRIα (MAR-1) has been suggested to represent a moDC marker. Whether moDCs in the inflamed CNS express MAR-1 has so far not been analyzed (65). Perhaps a more functional distinction should be drawn on the level of relevance for the inflammatory process to persist. CNS-infiltrating myeloid cells with DC-like morphology express MHCII, CD40, and CD86, all of which have critical roles in multiple inflammatory models (66). The CD86/CD28 interaction between T cells and APCs is of critical importance for T cell activation. Furthermore, the CD40/CD40L interaction induces a maturation pathway within the inflamed CNS, resulting in further costimulatory capabilities and proinflammatory cytokine expression (67, 68). The levels of CD40 on monocyte-derived cells in the inflamed CNS are variable but generally not as high as on classical DCs.

After activation, inflammatory macrophages can not only express a wide range of inflammatory cytokines but also oxygen-based chemically reactive molecules involved in host defense. The route an activated macrophage takes depends largely on the T cell and/or NK cell-derived cytokines present during their activation. For example, activation in the presence of LPS and IFN-γ leads to a “classical” activation (often called “M1”), resulting in secretion of high levels of TNF-α, iNOS, IL-1, IL-6, and IL-12. Conversely, activation of the same cells in the presence of IL-4 and IL-10 will result in rapid upregulation of IL-10, production of Arginase-1, and upregulation of the mannose receptor CD206, generating a macrophage capable of suppressing T cell activity (called “M2”) (69). This intracellular divergence in phenotype illustrates that an apparently similar cell expressing F4/80, CD64, CD11b, MHCII, and CD11c on its surface may, in fact, differ greatly in its function. Indeed, markers identifying both M1 and M2 macrophage populations have been shown synergistically in CNS biopsies obtained from MS patients. CD40, CD64, CD86, and CD32, mannose receptor and CD163 were co-expressed in the large majority of foamy macrophages found in lesional CNS (70). Therefore, surface characterization of inflammatory macrophages would appear insufficient and may mask different macrophage populations in direct opposition to each other, depending on the type of inflammation taking place. Generally in the steady state, tissue macrophages display an “M2-like” phenotype and are critical for tissue homeostasis. Interestingly, in a model of spinal cord injury, it was shown that M2 macrophages (CD11b^+^F4/80^+^CX_3_CR1^hi^Ly6C^lo^) are beneficial and promote recovery (62).

## Conclusion

Under steady state conditions, site specific and phenotypic characteristics exist to distinguish between microglia and other CNS-associated macrophages. As with almost all innate and adaptive immune cell types, consensus with respect to nomenclature in CNS-resident versus CNS-infiltrating myeloid cells has not been effectively reached under inflammatory conditions. The advent of microarray technology and next generation sequencing will serve to provide more useful ways to distinguish between these two apparently similar, yet ever more functionally diverse cell types. An ever-increasing variety of previously unappreciated, and non-immune homeostatic functions performed by macrophages are now beginning to emerge, making a more detailed separation of these cell types highly desirable (71). Ultimately, better characterization and dissection of the various myeloid cells in an inflamed brain will help deciphering the specialized functions of the different members of the MPS in pathological conditions.

## Conflict of Interest Statement

The authors declare that the research was conducted in the absence of any commercial or financial relationships that could be construed as a potential conflict of interest.
